# The “STOP Pain” Questionnaire: using the Plan-Do-Study-Act model to implement a patient-family preferences-informed questionnaire into a pediatric transitional pain clinic

**DOI:** 10.1186/s41687-022-00520-4

**Published:** 2022-11-29

**Authors:** Catherine Stratton, Jennifer Tyrrell, Rachel Goren, Chitra Lalloo, Lisa Isaac

**Affiliations:** 1 Department of Anesthesia and Pain Medicine, The Hospital for Sick Children, 555 University Ave, M5G 1X8 Toronto, ON Canada; 2 Lawrence S. Bloomberg Faculty of Nursing, University of Toronto, 155 College St, Suite 130, M5T 1P8 Toronto, ON Canada; 3 Institute for Health Policy, Management & Evaluation, University of Toronto, 155 College St 4th Floor, M5T 3M6 Toronto, ON Canada; 4 Temerty Faculty of Medicine, University of Toronto, Medical Sciences Building, 1 King’s College Cir, M5S 1A8 Toronto, ON Canada

**Keywords:** Transitional pain, Pain assessment, Pediatric pain, Patient-engagement, Questionnaire development, Quality improvement

## Abstract

**Background:**

Patient engagement is an important tool for quality improvement (QI) and optimizing the uptake of research findings. The Plan-Do-Study-Act (PDSA) model is a QI tool that encourages ongoing evaluation of clinical care, thus improving various aspects of patient care. Ascertaining pediatric patient priorities for a pain questionnaire in the post-acute, or transitional pain, setting is important to guide clinical care since active engagement with the population of interest can optimize uptake. We used the PDSA model to adapt a chronic pain questionnaire for the pediatric transitional pain setting to reflect pediatric patient and parent/guardian preferences and to form an example of how the PDSA model can be used to improve clinical care through patient engagement.

**Methods:**

This project employed the PDSA model to adapt the pediatric Ontario Chronic Pain Questionnaire for use in the pediatric Transitional Pain Service (pedTPS) setting. Plan: Following reviewing the Ontario Chronic Pain Questionnaire and literature on pain questionnaire development, goal-based questions, questions on pain location, relevant Patient-Reported Outcomes Measurement Information System (PROMIS^®^) measures and the Pain Catastrophizing Scale, child (PCS-C) and parent (PCS-P), informed the questionnaire. Do: The questionnaire and a satisfaction survey were sent to patients and families through Research Electronic Data Capture (REDCap™). Study: Results from the satisfaction survey were analyzed. Act: Using descriptive statistics employing ordinal mixed-models with random effects, ANOVA, and double-blinded qualitative thematic coding, questionnaire preferences were analyzed and the questionnaire was adapted accordingly before implementation into the (pedTPS).

**Results:**

Eighty-eight questionnaires and satisfaction surveys were analyzed from 69 respondents (32 patients; 37 parents/guardians). Sixty-six (75.00%) surveys indicated satisfaction with the questionnaire. A combined 77 (87.50%) “strongly agreed” (25/88) or “agreed” (52/88) that the questionnaire language was clear. The application of suggested changes to the questionnaire resulted in four versions across the project timeline, which reflected patient and parent/guardian preferences for questions that reflect the themes, “Story”; “Time-Optimal”; and “Pertinent” (“STOP”). There were no statistically significant differences in satisfaction across the versions due to sample size.

**Conclusion:**

Most respondents were satisfied with the questionnaire and prefer “STOP” questions. Future studies will focus on testing the questionnaire for validity and reliability across pedTPS populations.

**Supplementary information:**

The online version contains supplementary material available at 10.1186/s41687-022-00520-4.

## Introduction

### Background and scientific justification

Chronic pain is a serious pediatric health issue, affecting up to 38% of children [1]. Those with difficult to manage pain are at higher risk for multiple physical and psychological complications as well as decreased functionality [2–5]. Surgery and acute pain are predictors for long-term opioid use and developing chronic pain, which refers to pain that persists after three months and is associated with considerable functional disability and/or emotional distress [6–9]. The SickKids pediatric Transitional Pain Service (pedTPS) at the Hospital for Sick Children (SickKids) in Toronto, Ontario, to our knowledge, is the first pediatric clinic of its kind in North America. It provides pain consultation for children during peri-hospitalization care to limit the risk of and to “stop” pain before it becomes persistent or chronic [6]. The pedTPS is staffed by a team of anesthesiologists and advanced practice nurses, with consultation from pain psychologists, physical therapists, and occupational therapists, who work collaboratively to transition the patient from a major medical event to their regular routine, while addressing their transitional pain using a biopsychosocial model of pain. Referral to the pedTPS occurs preoperatively for patients with increased risk for acute pain or for developing persistent pain, such as child preoperative anxiety, pain unpleasantness, anxiety sensitivity [10], catastrophizing [11], postoperative pain [12], pain medication use [13, 14], and/or functional disability [14]. Parental factors, such as parental anxiety [15], high parental pain catastrophizing [16, 17], and/or surgical factors, such as an especially painful procedure, (e.g., scoliosis surgery) may be associated with ongoing severe pain [16–19] and functional disability [15]. Postoperative referral occurs for patients with high pain or high opioid use in hospital [15, 20]. Despite the integral role that transitional pain management plays in limiting the development of chronic or persistent pain, no standard pain assessment questionnaire exists to assess pain in the pediatric transitional pain setting. Therefore, our team sought to adapt a pain assessment questionnaire for this setting.

An important element of pain management is establishing individualized goals with patients concerning their recovery, though this remains a gap in pain questionnaire development [21]. In fact, mismatched expectations for pain assessment and management have been linked to poorer patient outcomes [22]. Several self-reported pain assessment scales are used in the clinical setting that are concerned with the intensity of pain, such as the visual analogue scale (VAS) and the Numeric Rating Scale (NRS) [23]. Validity for such metrics have been confirmed through multiple studies, showing criterion and construct validity, as well as reliability and responsiveness [24–26]. Conversely, some pain intensity measures also reflect pain interference and pain unpleasantness [27] with the NRS reflecting pain intensity more rigorously [19], while intensity still being influenced by other biopsychosocial factors including parental protective behaviours and disease activity [28]. Other assessment instruments are used to ascertain the nature of patients’ pain and the impact it has on patients’ lives, such as the Pediatric Pain Questionnaire (PPQ) [29]. Pain assessment has been criticized in the literature for its failure to consider the subjectivity of pain, differences in recovery goals between healthcare providers and patients, and the complexities of pain, such as patients who might experience different levels of pain in different parts of the body [4, 29, 30]. There is disagreement across healthcare providers and patient populations regarding which tool(s) to employ and efforts have been made to identify the suitability of specific scales for various adult patient populations [31, 32]. For instance, some previous studies have assessed adult patient preferences of various pain measurements [31–33]. The studies identified to date have not focused on the priorities for pain assessment among pediatric patients, provided a platform for patients to specify what information they want collected about their pain, or explained how these preferences could be implemented. Accurate and effective pain assessment is critical since the provider’s understanding of the patient’s pain will inform a treatment plan [23].

### The Plan-Do-Study-Act model

The Plan-Do-Study-Act (PDSA) model is a quality improvement (QI) tool that involves testing and implementing changes in a gradual manner for the improvement of a product or process, such as a pain assessment questionnaire [34]. PDSA cycles allow researchers to observe changes over time and assess which changes are worthy of implementation [34].

The PDSA model has been used in studies aimed at improving pain assessment [35] with several focusing on pediatric populations [36, 37]. The PDSA methodology has been used to incorporate patient feedback in an older patient rehabilitation setting [38]. This QI project used the PDSA model to assess the implementation of a questionnaire that is validated in the pediatric chronic pain setting into the pediatric transitional pain setting.

### Objectives

The primary objective was to use the PDSA model to adapt a chronic pain questionnaire for implementation in the pedTPS setting by incorporating patient and family feedback. The secondary objective was to form an example of how the PDSA model can be applied for the improvement of clinical care through patient engagement which could be applied in other clinical contexts.

## Methods

### Project design

**Fig. 1:**
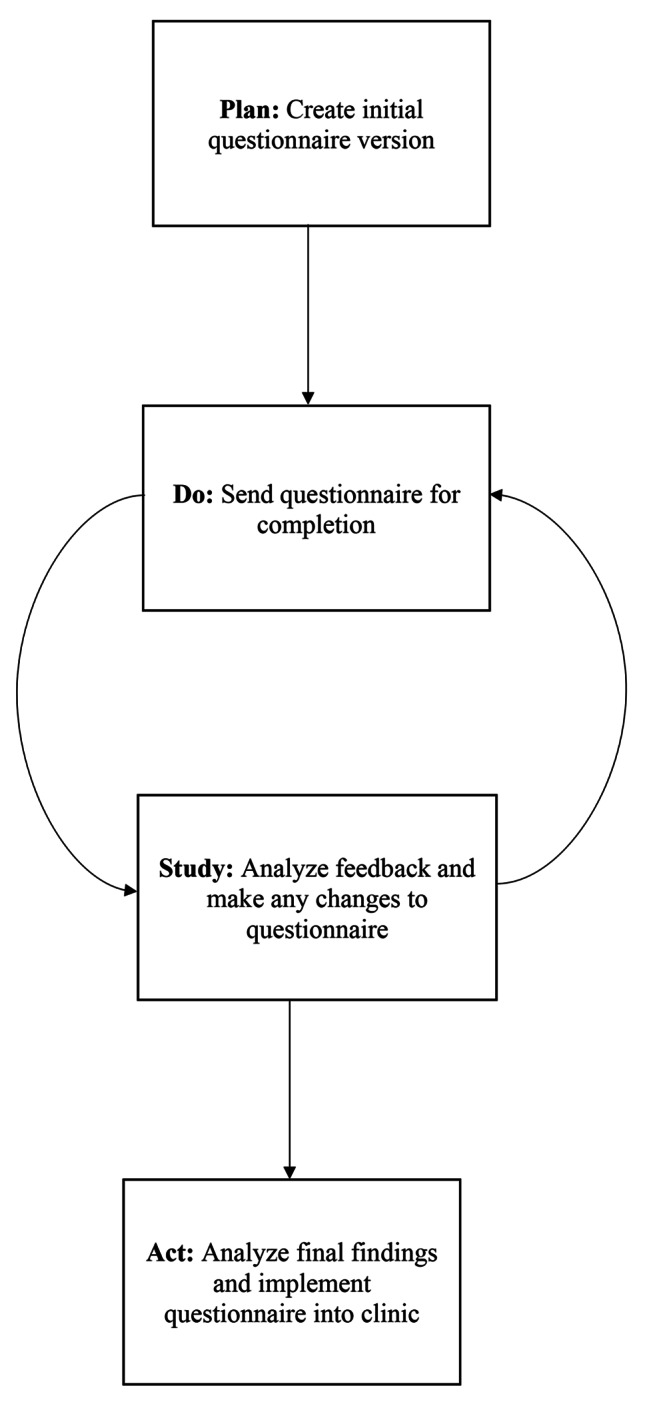
Plan-Do-Study-Act Cycle

By employing the PDSA model, this QI project involved adapting the mixed-method pain questionnaire used for the Pediatric Chronic Pain Registry (from the Ontario Chronic Pain Care Network) [39] to be used for pedTPS clinic visits, and then to seek feedback through a post-questionnaire satisfaction survey from patients and their parents/guardians to ascertain their priorities in the assessment of their transitional pain (Fig. 1.). The suggestions made in the satisfaction survey were reviewed and if deemed relevant to the patient population more broadly, the recommendation was adopted. This process allowed investigators to attempt to improve the assessment questionnaire and to determine changes to satisfaction over time. Feasibility was operationally assessed by patient and family satisfaction rate, completion rate, and time for completion.

### Ethics

This project was approved by the Quality Improvement Projects Board (QIPB) of the Hospital for Sick Children. Traditional formal consent was not required as QI projects are rooted in program evaluation and no experimentation is involved in the project. Further, patients would be completing the questionnaire for the clinic visit regardless of this project. The Research Ethics Board (REB) and QIPB co-developed this policy for QI projects with the understanding that care optimization is central to QI. The satisfaction survey automatically followed the questionnaire with no need for a separate link. Respondents had the option to leave the satisfaction survey blank or to exit Research Electronic Data Capture (REDCap™) at any time. It was also specified that care was not conditional on their responses to the satisfaction survey.

## Procedures

### Plan

With the understanding that there is no validated questionnaire for the pedTPS setting, and the understanding that a fundamental goal of pedTPS is to prevent the development of chronic pain, investigators decided that a reasonable starting point for forming a questionnaire for the pedTPS would be to review existing questionnaires for chronic pain. The Director of the pedTPS and a pedTPS nurse practitioner reviewed the Ontario Chronic Pain Care Network Chronic Pain Registry [39] and agreed upon which pain-related scales in this chronic pain registry would be appropriate for gathering clinically important information in the context of the pedTPS. It was decided that a questionnaire for assessing pain in the pedTPS setting adapted from the Chronic Pain Registry should include patient and parent-/guardian-proxy metrics for pain location, duration, and intensity, relevant Patient-Reported Outcomes Measurement Information System (PROMIS^®^) metrics [40] (Numeric Rating Scale v1.0 – Pediatric Pain Intensity 1a, Pediatric Short Form v1.0 – Sleep Disturbance 4a, Pediatric Short Form v2.0 – Pain Interference 8a, Pediatric Short Form v2.0 – Anxiety 8a, Pediatric Short Form v2.0 – Depressive Symptoms 8a, Parent Proxy Short Form v1.0 – Sleep Disturbance 4a, Parent Proxy Short Form v2.0 – Pain Interference 8a, Parent Proxy Short Form v2.0 – Anxiety 8a, Parent Proxy Short Form v2.0 – Depressive Symptoms 6a), and the Pain Catastrophizing Scales, child (PCS-C) and parent (PCS-P) [41].

While these pain-related scales from the Chronic Pain Registry were included in the proposed adapted questionnaire for the pedTPS setting, a targeted literature search was completed to identify gaps in patient and family input into questionnaire development as to propose a questionnaire for the pedTPS that is patient-informed. Common themes from the review were that qualitative, open-ended questions provide more robust patient information since the structure allows patients to share their particular concerns, fears, and goals for treatment [42–45]. With this knowledge, qualitative questions framed around the patient’s pain “story” were incorporated into the questions to ascertain patients’ and their parents’/guardians’ particular concerns and goals.

Since this project involved the adaptation and implementation of a questionnaire, investigators also developed a post-questionnaire satisfaction survey to receive feedback and improve the questionnaire.

### Do

The proposed questionnaire and survey were sent electronically to patients with separate questionnaire links for patients and parent(s)/guardian(s) during the week before their pedTPS clinic appointment in REDCap™. Participants received up to two electronic reminders before their appointment as needed. Those who did not complete the questionnaire before the appointment were given the opportunity to complete the questionnaire in the clinic on an iPad. The satisfaction survey was collected after the questionnaire and was completely voluntary. Data were collected from July 29, 2019 to February 13, 2020.

### Study

All questionnaires were reviewed. Completeness as well as the reported satisfaction and clarity scores were recorded. The responses to the open-ended, qualitative questions on recommendations to improve the questionnaire were analyzed and coded by two investigators (CS, RG) for emerging themes in codebooks. Categories for respondents’ comments regarding what to remove or add to the questionnaire were established *a priori* by reviewing the various pain-related scores being used. Disagreements were resolved through consensus. The reported descriptive statistics reflect the final categorization of comments by the investigators following their independent coding. The questionnaire content itself was reviewed in the patient’s appointment, but not considered for analysis in this project. Throughout the process, recommendations for changes to the questionnaire that were relevant to the patient population were implemented, cycling between the *Do* and *Study* stages until enrollment ended.

### Act

After the project was completed, a comparative data analysis of the satisfaction survey was conducted to ascertain which version of the questionnaire was most acceptable to the pediatric transitional pain population. While initially investigators had delivered the questionnaire to patient-families for all appointments, a schedule for questionnaire delivery, based around significant milestones (e.g., pre-surgical assessment, initial post-surgical assessment) was devised upon the completion of the project. PedTPS staff were trained about how to administer the questionnaire moving forward, ensuring a smooth implementation into the clinical setting.

## Patient population

A convenience sample was sought from all patients of the pedTPS who met the inclusion criteria below.

### Inclusion criteria

The questionnaire was sent electronically to all patients being treated in the pedTPS who were fluent in English, eight years or older, and able to respond to the questionnaire. The questionnaire was also sent to parents/guardians who were fluent in English. Parent proxy forms are an accepted practice in pain medicine to help verify reporting, fill gaps in completion as well as provide information relevant to the pain experience that is not always captured with pediatric patients. For example, parental catastrophizing is separate from the child, but relevant to their pain experience [46].

### Exclusion criteria

In keeping with PROMIS^®^ standards, the questionnaire was not sent to patients who were developmentally younger than eight years old or to patients or parents/guardians who were known to the pedTPS as not being fluent in English at this level regardless of developmental age [47].

## Materials and instruments

The project employed the newly developed pedTPS questionnaire. The REDCap™ questionnaire was sent electronically prior to visits in the pedTPS clinic. Additionally, a satisfaction survey was designed to assess the questionnaire. The satisfaction survey asked respondents to respond to the prompts, “The questionnaire captured what I think is important about my/my child’s pain” and “The language in the questionnaire was clear”, using a Likert-scale (5 = “Strongly agree”, 4 = “Agree”, 3 = “Neither agree nor disagree”, 2 = “Disagree”, 1 = “Strongly disagree”). Secondly, respondents were asked to use free-text responses to make suggestions as to what they would have removed or added (if anything) to the questionnaire. The questionnaire versions, which were developed based on participant-led iterative modifications, were explored to determine if this process affected survey completion rate, survey comprehensiveness and clarity.

## Data analysis

### Quantitative analyses

Descriptive statistics were used to measure satisfaction with the questionnaire. Further, satisfaction was compared across versions to determine if there was a superior version from the patient-family perspective.

We employed an ordinal mixed-model approach to accommodate both fixed and random effects. Fixed effects are factors that are unchanged, such as patient sex, while random effects are factors that could change across different respondents and different occasions of completing the questionnaire by the same respondent (e.g., their reason for referral to the pTPS, their pain symptoms and experiences, etc.) [48]. Consistent with QI principles, we asked patients only to participate if they already had an appointment at the pTPS and would therefore be completing the questionnaire as part of the clinical assessment. This does mean, however, that some respondents only provided feedback once while others responded twice. For the latter group, there is a comparison in satisfaction to be made across questionnaire versions. A mixed-model is well-suited for this scenario since it can take multiple measurements from a single respondent into account [48]. Further, a mixed-model can accommodate for differences in the amount of data reported by each participant [48]. ANOVA was used to confirm the findings from the mixed-model. A weighted Kappa coefficient was calculated for measuring inter-rater reliability for the qualitative theme coding described below [49].

### Qualitative analysis

In addition to the quantitative feedback, the satisfaction survey asked respondents to share, in open-text style, which, if any, sections or questions they would have removed or added to the questionnaire. A thematic coding analysis was conducted using codebooks to categorize the most common preferences for the questionnaire.

## Results

### Survey completion rate, respondent characteristics, and overall satisfaction and clarity

#### Completion and characteristics

Over the project period, 184 questionnaires were sent electronically to patients and parents/guardians meeting the inclusion criteria. Ninety questionnaires (90/184, 48.91%) were fully completed, though since one participant completed the questionnaire and survey thrice, the third questionnaire was removed from analysis, resulting in 89 completed questionnaires. One respondent did not respond to the satisfaction survey so 88 surveys (88/89, 98.88%) were included in analysis. Among those sent the questionnaire more than once, 88.68% completed it at least once. Since 19 respondents completed the questionnaire a second time, the 88 surveys analyzed belonged to 69 respondents.

Of the 69 individual respondents, 32 (32/69, 46.38%) were patients and 37 (37/69, 53.62%) were parents/guardians. Of the 88 survey responses, 42 (42/88, 47.73%) were patients and 46 (46/88, 52.27%) were parents/guardians. Paired patient-parent dyads accounted for 56 (56/88, 63.64%) of the 88 completed questionnaires whereas 32 questionnaires (32/88, 36.36%) were responded to by either a patient or a parent/guardian, but not both. Patients seen in the pedTPS are aged 8 to 18 years old and the ages of the patient respondents ranged from 10 to 17 years old (overall: mean (M) = 14.55 years, standard deviation (SD) = 2.33; males: M = 15.44 years, SD = 1.85; females: M = 13.31 years, SD = 2.50). One male respondent did not disclose their age. The sample appears somewhat reflective of the patient population who did not respond to the questionnaire, with greater representation of males where 19 (19/32, 59.38%) patient respondents were male and 13 (13/32, 40.63%) patient respondents were female.

#### Decision-making in survey versions and resulting final version

**Fig. 2:**
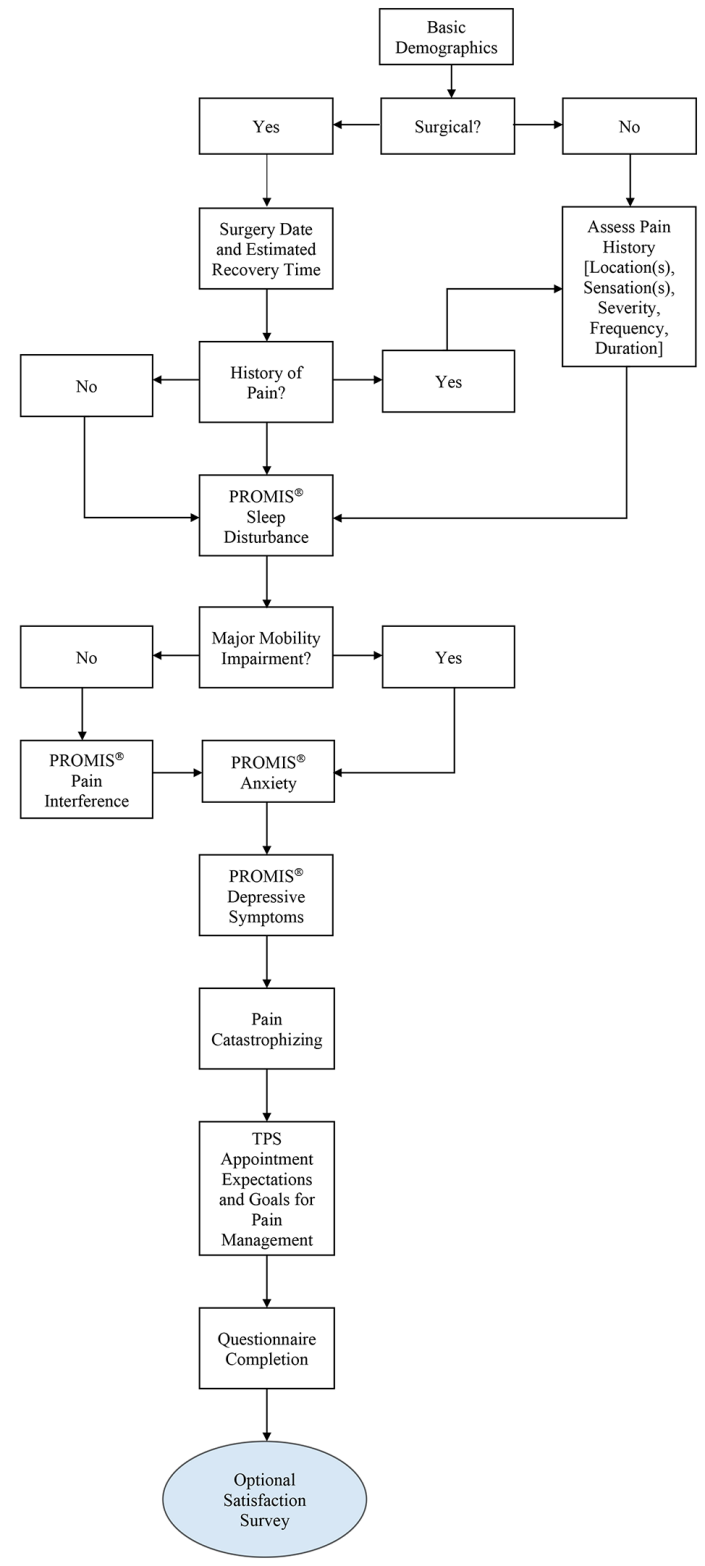
Final Questionnaire Pathway

The project resulted in four versions whose details including the date, the number of completed questionnaires of each version, and the substantive change(s) defining each version is summarized (Table 1.). The decision to make a revision to the questionnaire was informed by repeated concerns in respondents’ suggestions and agreement across investigators that the suggestions would be pertinent to most patients, rather than patient-specific circumstances which would be resolved individually. A change was only considered after a minimum of three respondents raised the same thematic concern. While seemingly like a small number of respondents to merit a change to the questionnaire, the pedTPS population is quite heterogenous while the sub-groups (e.g., scoliosis, complex care, Nuss pectus repair, etc.) within the broader patient population are relatively homogenous. We wanted any changes made to the questionnaire to allow for customization to improve the experience of patients by limiting questionnaire burden and by considering specific clinical circumstances. Some of these changes were ethically based by adapting the questionnaire pathway to skip over some questionnaires that were irrelevant and emotionally triggering to some patients. This outlines an example of how we assessed a meaningful change. A pathway to the final version is outlined which reflects these amendments (Fig. 2.). Since there have been no statistically significant differences across the questionnaire versions, and the majority of respondents report satisfaction with the questionnaire, the fourth and final version of the questionnaire has been adopted as it is the most thorough and incorporates the most patient feedback.

**Table 1:** Versions of the Questionnaire

Questionnaire Version	Date of Implementation	Number of Completed Questionnaires Associated with this Version	Defining Characteristic/Change in Version
1	July 29, 2019	11	Original document
2	September 3, 2019	45	Parents/guardians answering the goal-based questions which were only asked of patients previously
3	December 3, 2019	15	Added PROMIS^®^ Pediatric and Parent Proxy Depressive Symptoms scales and question about current pharmaceutical/medication use for pain management
4	January 14, 2020	17	Question about mobility impairment is added in order for patients unable to walk/run to skip over the PROMIS^®^ Pediatric and Parent Proxy Pain Interference scales

The first questionnaire version asked goal-based questions of the patients, but not of their parents/guardians to focus on patient goals without parental influence. After parents/guardians expressed the desire for a platform to discuss their goals for their child, these questions were added into the parent/guardian proxy form in the second version of the questionnaire. The respondent who had first made this suggestion completed the questionnaire after this amendment was made and changed their satisfaction rating from 2.00 to 5.00.

The third questionnaire version reflected the addition of the PROMIS^®^ Pediatric and Parent Proxy Depressive Symptoms scales and a question about current pharmaceutical treatments for pain (if any). Several patients and parents/guardians had indicated interest in a question that would inquire about patients’ emotional state and the pedTPS physician felt it was important that this information be measured objectively. Through consultation with two of the pedTPS psychologists, investigators implemented the PROMIS^®^ Pediatric and Parent Proxy Depressive Symptoms scales.

The final version of the questionnaire included an amendment to the questionnaire pathway. By adding in the question, “Do you/does your child have a major mobility impairment?,” those who responded, “yes” would bypass the PROMIS^®^ Pediatric and Parent Proxy Pain Interference scales. This change was applied to make the questionnaire more efficient for respondents with mobility issues and address repeated concerns raised by patients and parents/guardians about the relevance of questions relating to effects of pain on mobility in this setting for patients with a primary mobility impairment.

#### Satisfaction and clarity – effects of questionnaire version and questionnaire completion time

Sixty-six survey results (66/88, 75.00%) suggested that survey respondents felt the questionnaire captured important information about their/their child’s pain while only four survey results (4/88, 4.55%) reflected respondents who disagreed with this conclusion. The remaining responses (18/88, 20.45%) were that of respondents who neither agreed nor disagreed. Among these 66 survey responses indicating satisfaction, 30 (30/66, 45.45%) were from patients and 36 (36/66, 54.55%) were from parents/guardians. These proportions represented 71.43% (30/42) and 78.26% (36/46) of total responses belonging to these respective groups, suggesting that a greater proportion of parents/guardians than patients were satisfied with the questionnaire. Of the 88 surveys, a combined 77 (87.50%) “strongly agreed” (25/88, 28.41%) and “agreed” (52/88, 59.09%) that the language in the questionnaire was clear. Among these 77 survey responses, 35 were from patients and 42 were from parents/guardians. These responses represented 83.33% (35/42) and 91.30% (42/46) of the total survey responses belonging to these respective groups, suggesting that a greater proportion of parents/guardians than patients felt that the questionnaire’s language was clear. Only two survey responses suggested that respondents “disagreed” (1/88, 1.14%) or “strongly disagreed” (1/88, 1.14%) that the language was clear. The mean satisfaction scores for patients and parents/guardians were 3.88 and 3.87 out of 5.00 respectively, while the mean clarity scores for patients and parents/guardians were 4.17 and 4.09 out of 5.00. The combined mean satisfaction score for Version 4 was 4.00 out of 5.00 and the combined mean clarity score for Version 4 was 4.38 out of 5.00. The complete record of patient- and parent/guardian- respondents’ satisfaction and clarity ratings are reported in Table 2.

**Table 2:** *Response Ratings of Questionnaire Satisfaction and Clarity (Aggregate Data Across Versions)

Response	Satisfaction (n = 88) No. (%)	Response Breakdown		Clarity (n = 88) No. (%)	Response Breakdown	
		**Patient Responses** **(n = 42)** **No. (%)**	**Parent/Guardian Responses** **(n = 46)** **No. (%)**		**Patient Responses** **(n = 42)** **No. (%)**	**Parent/Guardian Responses** **(n = 46)** **No. (%)**
Strongly Agree	18 (20.45)	9 (21.43)	9 (19.57)	25 (28.41)	15 (35.71)	10 (21.74)
Agree	48 (54.55)	21 (50.00)	27 (58.70)	52 (59.09)	20 (47.62)	32 (69.57)
Neither Agree Nor Disagree	18 (20.45)	11 (26.19)	7 (15.22)	9 (10.23)	6 (14.29)	3 (6.52)
Disagree	2 (2.27)	0 (0.00)	2 (4.35)	1 (1.14)	1 (2.38)	0 (0.00)
Strongly Disagree	2 (2.27)	1 (2.38)	1 (2.17)	1 (1.14)	0 (0.00)	1 (2.17)

*Please note that due to rounding, the totals may not add up to 100.00% exactly.

The mean completion time of the questionnaire was 10 minutes, 14 seconds and the mode time of the questionnaire was ten minutes. Only six surveys (6/88, 6.82%) took respondents more than 15 min to complete the questionnaire. The results suggest that those who completed the survey in less time were more satisfied and found the questionnaire clearer. While changes in satisfaction and clarity were observed across questionnaire versions and completion times, the mixed-model and ANOVA revealed that these differences were not statistically significant.

### Qualitative analysis: categorizing patient-family feedback

#### Categorization of comments in the satisfaction survey

The full list of categories is reported in the Supplementary Material. It should be noted that one respondent elected to leave the open-response questions on the survey blank while some other respondents made more than one comment and so the sum of comments does not equal 88. The calculated weighted Kappa coefficient demonstrated strong inter-rater reliability (weighted Kappa = 0.94, 95% CI = 0.929–0.958, p<0.001).

#### Suggestions regarding removing questionnaire elements

Ninety-one comments were made about what questionnaire elements to remove, 43 (43/91, 47.25%) made by patients and 48 (48/91, 52.75%) by parents/guardians. The most frequent response to the prompt regarding removing questionnaire elements was, “No Suggested Changes” with 57 (57/91, 62.64%) comments. This category represented 58.14% (25/43) and 66.67% (32/48) of patients’ and parents’/guardians’ comments, respectively. Eleven (11/91, 12.09%) comments were categorized as, “Tailor (Mobility and Pain Interference)” since these suggestions were about removing questions regarding mobility or other domains of life which are impeded by pain. In these comments, respondents expressed concern that the questions were too broad and did not encapsulate specific clinical circumstances, such as imposed post-surgical restrictions on mobility or school attendance. Among these comments, five were made by patients and the remaining six were made by parents/guardians, representing 11.63% and 12.50% of these groups’ comments. Of note, no respondents suggested the removal of the open-ended questions. The summation of all respondents’ comments is reported in Table 3.

**Table 3:** *Distribution of Survey Comments on what to Remove from the Questionnaire

Category	If any, which sections of questions do you think could be removed from the questionnaire? (n = 91) No. (%)	Response Breakdown	
		**Patient Responses** **(n = 43)** **No. (%)**	**Parent/Guardian Responses** **(n = 48)** **No. (%)**
No Suggested Changes	57 (62.64)	25 (58.14)	32 (66.67)
Tailor Questionnaire to specific needs (Mobility and Pain Interference)	11 (12.09)	5 (11.63)	6 (12.50)
Repetition and Redundancy	6 (6.60)	4 (9.30)	2 (4.17)
Tailor Questionnaire to specific needs (General)	5 (5.49)	3 (6.98)	2 (4.17)
Unsure Respondent	5 (5.49)	2 (4.65)	3 (6.25)
Tailor Questionnaire to specific needs (Sleep)	3 (3.30)	2 (4.65)	1 (2.08)
Pain Description and Measurement	1 (1.10)	1 (2.33)	0 (0.00)
Typographical Revisions	1 (1.10)	0 (0.00)	1 (2.08)
Demographics	1 (1.10)	0 (0.00)	1 (2.08)
Satisfaction Survey	1 (1.10)	1 (2.33)	0 (0.00)

*Please note that due to rounding, the totals may not add up to 100.00% exactly.

#### Suggestions regarding adding questionnaire elements

Ninety-three comments were made about what questionnaire elements to add, 44 (44/93, 47.31%) made by patients and 49 (49/93, 52.69%) by parents/guardians. The most common response to the prompt regarding adding questionnaire elements was, “No Suggested Changes” with 46 (46/93, 49.46%) comments. Among these comments, 23 were made by patients and parents/guardians each, representing 52.27% (23/44) and 46.94% (23/49) of these groups’ comments. Another four (4/93, 4.30%) responses suggested adding elements to the questionnaire which they might have missed or overlooked while completing the questionnaire as their suggested additions were already reflected in the questionnaire. Adding more questions regarding “Medical History” and “Activities of Daily Life” were reflected in seven (7/93, 7.53%) and eight (8/93, 8.60%) of the suggestions, respectively. Adding further questions about the patient’s emotional state accounted for five (5.38%) of comments. The summation of all respondents’ comments is reported in Table 4.

**Table 4:** *Distribution of Survey Comments on what to Add from the Questionnaire

Category	If any, what other questions do you wish had been asked? (n = 93) No. (%)	Response Breakdown	
		**Patient Responses** **(n = 44)** **No. (%)**	**Parent/Guardian Responses** **(n = 49)** **No. (%)**
No Suggested Changes	46 (49.46)	23 (52.27)	23 (46.94)
Pain Description and Measurement	10 (10.75)	5 (11.36)	5 (10.20)
Activities of Daily Life	8 (8.60)	4 (9.09)	4 (8.16)
Unsure Respondent	8 (8.60)	7 (15.90)	1 (2.04)
Medical History	7 (7.53)	1 (2.27)	6 (12.24)
Emotional State	5 (5.38)	3 (6.82)	2 (4.08)
Tailor Questionnaire to specific needs (General)	4 (4.30)	0 (00.00)	4 (8.16)
Suggestion is already reflected in the Questionnaire	4 (4.30)	1 (2.27)	3 (6.12)
Opposed to Questionnaires in General for Pain Assessment	1 (1.08)	0 (0.00)	1 (2.04)

*Please note that due to rounding, the totals may not add up to 100.00% exactly.

## Discussion

### Clinical significance

This is, to our knowledge, the first pain assessment questionnaire for the pediatric transitional pain setting and the first pain questionnaire to use the PDSA model to incorporate pediatric patient-family feedback for a pain assessment questionnaire in any setting. These findings reveal pain assessment questionnaires that lack free-response questions might not be sufficient in capturing patient goals. This project successfully proposed a pain assessment questionnaire that reflects patient-family preferences that could be implemented into the pedTPS setting. Involving patients and families in questionnaire development may improve “pain readiness” which refers to the adoption of pain management suggestions that are vitally important for patient outcomes [50].

### The standardized tool dilemma

The substantial comments about making the questionnaire more tailored to individual patients introduced one of the challenges with using standardized scales, such as PROMIS^®^ instruments. As a validated tool, clinicians using PROMIS^®^ instruments cannot alter individual questions within the instrument. The PROMIS^®^ questionnaires were designed to determine pain-related outcomes in children with chronic pain and not acute or transitional pain [51] which may explain why some questions were irrelevant to some patients and parents/guardians.

Further, investigators did not want to remove the PROMIS^®^ Pediatric Pain Interference scale all together since the measure captures the impact of pain on physical functioning for mobile patients. Likewise, answering questions which are not relevant to the patient could also bias the questionnaire and result in a less precise t-score which would affect clinical decision-making. For example, if a patient cannot walk or run, and so selects responses to these questions at random (since they cannot be omitted or skipped), then the outcome t-score could be skewed, over- or underestimating the patient’s pain interference.

These conflicting factors were the basis for using the PROMIS^®^ Pediatric Pain Interference scale only if a patient has no major mobility issues. This amendment was indicative of how investigators implemented patient-family feedback while still ensuring the questionnaire would include provider-deemed necessary information.

### Factors impacting satisfaction and clarity

The high level of satisfaction among first-time questionnaire respondents was promising, although the few respondents who were not satisfied are indicative of how questionnaires will not appeal to everyone. Further, while not a statistically significant improvement, there was a greater proportion of respondents who were satisfied with the questionnaire and who felt it used clear language among those who completed it more than once. This finding is possibly due to familiarity with the questionnaire and having the opportunity to see the implemented improvements, which could empower patients and families knowing their feedback was utilized.

While clarity ratings remained quite high across all questionnaire versions, it is important to consider the implications of small changes in clarity across versions. Firstly, it exposes how incorporating patient feedback can be met with mixed reactions due to differing patient-family preferences. Further, it suggests that the addition of questions provides patient and parental respondents greater opportunity to discuss their preferences, but also may inhibit clarity by making the questionnaire longer and more complex. The final questionnaire version, however, illustrates that it is possible to have a questionnaire that addresses multiple preferences without inhibiting clarity.

The mean and mode completion times were approximately ten minutes. Excessive paperwork that asks for information beyond what is clinically necessary has been shown to be a greater burden than raw completion time among patients and families across medical specialties [52]. It was, therefore, a priority for this pain assessment questionnaire to be efficient without comprising comprehensiveness in order to optimize implementation. The lack of a statistically significant relationship between questionnaire completion time and clarity is evidence that decreasing questionnaire length and completion time does not yield greater confusion or suggest that respondents who completed the questionnaire in less time had rushed or were less engaged.

### Factors impacting completion rates

Duplicate opportunities to complete the questionnaire occurred only with in-person visits, which occurred for most new patients, but not for follow up visits, as per usual clinic practice. This may have improved the response rate, consistent with single-subject completion rates for web-based surveys [53].

The completion rates of the questionnaire and survey were lower than desired, but within the range of other patient-reported outcome questionnaires [53,54]. Factors such as negative previous experiences with completing questionnaires or the fact that patients and families could proceed with their appointment if the questionnaire is not completed, may yield lower completion rates in pre-appointment conducted questionnaires. Time required to complete the questionnaire may also influence overall completion rates, but likely not response rates.

It is interesting that 88.68% of those sent the questionnaire more than once completed it at least once, suggesting that persistence or reminding may be useful. Conversely, though, completion of second- or third-time questionnaires could have been impacted by the frequency of their conduction. If a patient and parent/guardian recently completed the questionnaire, they could be less inclined to complete it again since it may be perceived as burdensome and redundant if no significant changes in pain have occurred since the previous pedTPS appointment. This schedule is flexible and additional questionnaires can be delivered to patients and parents/guardians on an as-needed basis. This strategy aims to decrease unnecessary burden for patients and parents/guardians as well as improve the overall completion rate of the questionnaire in the pedTPS.

### Importance of goal-based questions

The overall high satisfaction rate and the addition of the free-response, qualitative questions suggest patient-family preference for using goal-based questions that enable patient partnership in care. Further, while some respondents suggested additional qualitative questions, no respondents suggested the omission of free-response questions. This is in stark contrast with the considerable number of recommendations to remove elements of the standardized PROMIS^®^ or entire PROMIS^®^ sections all together. These data shed light on the discussion regarding the importance of mixed-methodology in pain assessment for patients who prefer a conversational, goal-based approach to pain management.

These observations direct the major patient-family priorities identified:

1. **S**tory: Patients and families appreciate the opportunity to share their pain “story”, goals, and concerns for pain management, rather than respond to standardized questions which might not fully or accurately capture their priorities;
2. **T**ime-**O**ptimal: Less time spent by patients and parents/guardians on the questionnaire (without jeopardizing comprehensiveness) yields greater engagement. Clinicians should include solely medically necessary information;
3. **P**ertinent: To be included in the questionnaire, questions should be pertinent to the patient and their individual circumstances; repetitive or redundant questions decrease satisfaction and irrelevant questions decrease reliability of results.

### Limitations

One limitation, not unique to this questionnaire, is the fact that basic literacy skills in English are required to use this pain assessment questionnaire. Accordingly, non-English speakers and children who are not developmentally eight years or older could not actively participate and share their preferences. Some parents/guardians of children under the age of eight years old and/or who have an intellectual disability, who did have the opportunity to complete the questionnaire, shared in their clinical appointments that upon reviewing it, the proposed questionnaire did not encapsulate their concerns for their children.

### Future clinical and research directions

While the PDSA model was appropriate for the iterative nature of questionnaire development and feedback implementation, it did result in heterogenous and relatively small sample sizes of respondents to each questionnaire version. As such, after establishing these preliminary patient and family preferences, we will next conduct concept elicitation and cognitive debriefing interviews to confirm and/or adjust these preferences followed by a retest of the questionnaire with any changes incorporated in a larger sample from the pedTPS and other pediatric centres. This future work will help investigators to establish the reliability, validity, and generalizability of the questionnaire in this setting.

## Conclusion

This QI project introduced a novel application of the PDSA model which uses patient and family feedback to modify a pediatric chronic pain assessment questionnaire for the pediatric transitional pain setting. The incorporation of mixed-methodology, that is, the combination of standardized pain scales and free-response, goal-based questions, was acceptable across most respondents, though analysis indicated preference for the goal-based questions. Improving questionnaire feasibility is based in optimizing questionnaire length, using patient-/family-deemed clear language, ensuring sections are relevant to the patient or parent/guardian completing the questionnaire, and providing a platform for patients to share their goals for pain management.

## Electronic supplementary material

Below is the link to the electronic supplementary material.

Supplementary Material 1

## Data Availability

The datasets generated and/or analysed during the current study are not publicly available due it being patient provided information for quality improvement.

## References

[1] King S, Chamber C, Huguet A, MacNevin R, McGrath P, Parker L, MacDonald A (2011). The epidemiology of chronic pain in children and adolescents revisited: A systematic review. Pain.

[2] Åkerblom S, Perrin S, Fischer MR, McCracken LM (2017). The Impact of PTSD on Functioning in Patients Seeking Treatment for Chronic Pain and Validation of the Posttraumatic Diagnostic Scale. Int J Behav Med.

[3] Day M, Lang C, Newton-John T, Ehde D, Jensen M (2016). A content review of cognitive process measures used in pain research within adult populations. Eur J Pain.

[4] Haanpää ML, Gourlay GK, Kent JL et al (2010) Treatment considerations for patients with neuropathic pain and other medical comorbidities. Mayo Clin Proc. ;85(3 Suppl):S15–S25. 10.4065/mcp.2009.0645 10.4065/mcp.2009.0645 PMC2844009 20194144

[5] Wicksell RK, Kanstrup M, Kemani MK, Holmström L (2016). Pain Interference Mediates the Relationship between Pain and Functioning in Pediatric Chronic Pain. Front Psychol.

[6] Katz J, Weinrib A, Fashler SR et al The Toronto General Hospital Transitional Pain Service: development and implementation of a multidisciplinary program to prevent chronic postsurgical pain.J Pain Res.2015;(8):695–702. 10.2147/JPR.S91924 10.2147/JPR.S91924 PMC4610888 26508886

[7] Lavand’homme P (2017). Transition from acute to chronic pain after surgery. Pain.

[8] Nicholas M, Vlaeyen JWS, Rief W, Barke A, Aziz Q, Benoliel R, Cohen M, Evers S, Giamberardino MA, Goebel A, Korwisi B, Perrot S, Svensson P, Wang SJ, Treede RD ; IASP Taskforce for the Classification of Chronic Pain. The IASP classification of chronic pain for ICD-11: chronic primary pain.Pain. 2019 Jan; 160(1):28–37. 10.1097/j.pain.0000000000001390 10.1097/j.pain.0000000000001390 30586068

[9] Weinrib AZ, Burns LC, Mu A (2017). A case report on the treatment of complex chronic pain and opioid dependence by a multidisciplinary transitional pain service using the ACT Matrix and buprenorphine/naloxone. J Pain Res.

[10] Pagé MG, Stinson J, Campbell F, Isaac L, Katz J (2013). Identification of pain-related psychological risk factors for the development and maintenance of pediatric chronic postsurgical pain. J Pain Res.

[11] Birnie KA, Chambers CT, Spellman CM (2017). Mechanisms of distraction in acute pain perception and modulation. Pain.

[12] Batoz H, Semjen F, Bordes-Demolis M, Bénard A, Nouette-Gaulain K (2016). Chronic postsurgical pain in children: prevalence and risk factors. A prospective observational study. Br J Anaesth.

[13] Ocay DD, Li MMJ, Ingelmo P, Ouellet JA, Pagé MG, Ferland CE Predicting Acute Postoperative Pain Trajectories and Long-Term Outcomes of Adolescents after Spinal Fusion Surgery.Pain Res Manag. 2020;2020:9874739. Published 2020 Feb 24. 10.1155/2020/987473 10.1155/2020/9874739 PMC7060857 32184913

[14] Rosenbloom BN, Pagé MG, Isaac L (2019). Pediatric Chronic Postsurgical Pain And Functional Disability: A Prospective Study Of Risk Factors Up To One Year After Major Surgery. J Pain Res.

[15] Rosenbloom BN, Slepian PM, Pagé MG, Isaac L, Campbell F, Stinson J, Katz J (2021). Differential Risk Factor Profiles in the Prediction of General and Pain-Specific Functional Limitations 12 Months after Major Pediatric Surgery. Children.

[16] Rabbitts JA, Fisher E, Rosenbloom BN, Palermo TM (2017). Prevalence and Predictors of Chronic Postsurgical Pain in Children: A Systematic Review and Meta-Analysis. J Pain.

[17] Voepel-Lewis T, Caird MS, Tait AR (2017). A High Preoperative Pain and Symptom Profile Predicts Worse Pain Outcomes for Children After Spine Fusion Surgery. Anesth Analg.

[18] Chidambaran V, Ding L, Moore DL (2017). Predicting the pain continuum after adolescent idiopathic scoliosis surgery: A prospective cohort study. Eur J Pain.

[19] Connelly M, Fulmer RD, Prohaska J et al (2014) Predictors of postoperative pain trajectories in adolescent idiopathic scoliosis. Spine (Phila Pa 1976). ;39(3):E174-E181. https://doi.org10.1097/BRS.0000000000000099 10.1097/BRS.0000000000000099 24173016

[20] Harbaugh CM (2018). Persistent Opioid Use Among Pediatric Patients After Surgery. Pediatrics.

[21] Gardner T, Refshauge K, McAuley J et al (2016 Sep) Patient-led Goal Setting: A Pilot Study Investigating a Promising Approach for the Management of Chronic Low Back Pain. Spine 41(18):1405–1413. 10.1097/brs.0000000000001545 10.1097/BRS.0000000000001545 26937604

[22] Canada H (2020) Government of Canada. Canadian Pain Task Force Report: September 2020 - Canada.ca. https://www.canada.ca/en/health-canada/corporate/about-health-canada/public-engagement/external-advisory-bodies/canadian-pain-task-force/report-2020.html. Published November 6, Accessed July 14, 2021

[23] Hjermstaf M, Fayers P, Haugen D, Caraceni A, Hanks G, Loge J, Kaasa S (2011). Studies Comparing Numerical Rating Scales, Verbal Rating Scales, and Visual Analogue Scales for Assessment of Pain Intensity in Adults: A Systematic Literature Review. J Pain Symptom Manag.

[24] Birnie KA, Hundert AS, Lalloo C, Nguyen C, Stinson JN (2019). Recommendations for selection of self-report pain intensity measures in children and adolescents: a systematic review and quality assessment of measurement properties. Pain.

[25] Castarlenas E, Jensen MP, von Baeyer CL, Miró J (2017). Psychometric Properties of the Numerical Rating Scale to Assess Self-Reported Pain Intensity in Children and Adolescents. Clin J Pain.

[26] Miró J, de la Vega R, Gertz KJ, Thong ISK, Jensen MP, Engel JM (2019). Do Commonly Used Measures of Pain Intensity Only Reflect Pain Intensity in Youths With Bothersome Pain and a Physical Disability?. Front Pediatr.

[27] Thong ISK, Jensen MP, Miró J, Tan G (2018). The validity of pain intensity measures: what do the NRS, VAS, VRS, and FPS-R measure?. Scand J Pain.

[28] Murphy L, Rights JD, Ricciuto A, Church PC, Kohut SA (2020). Biopsychosocial Correlates of Presence and Intensity of Pain in Adolescents With Inflammatory Bowel Disease. Front Pediatr.

[29] Varni JW, Thompson KL, Hanson V (1987). The Varni/Thompson Pediatric Pain Questionnaire. I. Chronic musculoskeletal pain in juvenile rheumatoid arthritis. Pain.

[30] Leung L (2012). Pain catastrophizing: an updated review. Indian J Psychol Med.

[31] Jensen M, Johnson L, Gertz K, Galer B, Gammaitoni A (2013). The words patients use to describe chronic pain: implications for measuring pain quality. Pain.

[32] Karcioglu O, Topacoglu H, Dikme O, Dikme O (2018). A systematic review of the pain scales in adults: Which to use?. Am J Emerg Med.

[33] Clark P, Lavielle P, Martinez H (2003). Learning from pain scales: patient perspective. J Rheumatol.

[34] Quality SI, Tools R (2008) : Plan, Do, Study, Act (PDSA) cycles and the model for improvement. Quality, Service Improvement and Redesign Tools: Plan, Do, Study, Act (PDSA) Cycles and the Model for Improvement. NHS Improvement. Accessed at: https://www.england.nhs.uk/wp-content/uploads/2021/03/qsir-plan-do-study-act.pdf on December 23, 2021

[35] Emily Hurstak, Maria T, Chao K, Leonoudakis-Watts J, Pace (2019) Blue Walcer, and Barbara Wismer.The Journal of Alternative and Complementary Medicine. 10.1089/acm.2018.0398. .S78-S 10.1089/acm.2018.0398 30870021

[36] Sil S, Goldstein-Leever A, Travers C (2019). Enhancing Pain Assessment in Pediatric Sickle Cell Disease by Applying Quality Improvement Science. Clin Pract Pediatr Psychol.

[37] Lynch-Jordan AM, Kashikar-Zuck S, Crosby LE (2010). Applying quality improvement methods to implement a measurement system for chronic pain-related disability. J Pediatr Psychol.

[38] McGowan M, Reid B (2018) Using the Plan, Do, Study, Act cycle to enhance a patient feedback system for older adults. Br J Nurs. Sep 6;27(16):936–941. 10.12968/bjon.2018.27.16.936 10.12968/bjon.2018.27.16.936 30187794

[39] Manon Choinière R, Hovey N, Buckley I, Gilron C, Maye JS, Ware M (2018).

[40] HealthMeasures (2016) *PROMIS*^*®*^ Retrieved from http://www.nihpromis.org/

[41] Crombez G, Bijttebier P, Eccleston C, Mascagni T, Mertens G, Goubert L, Verstraeten K The child version of the pain catastrophizing scale (PCS-C): a preliminary validation.Pain. 2003Aug; 104(3):639–646. 10.1016/S0304-3959(03)00121 10.1016/S0304-3959(03)00121-0 12927636

[42] Geurts JW, Willems PC, Lockwood C, van Kleef M, Kleijnen J, Dirksen C (2017). Patient expectations for management of chronic non-cancer pain: A systematic review. Health Expect.

[43] Barlow T, Griffin D, Barlow D, Realpe A (2015). Patients’ decision making in total knee arthroplasty: a systematic review of qualitative research. Bone Joint Res.

[44] Erik Riiskjær J, Ammentorp P-E, Kofoed (October 2012) The value of open-ended questions in surveys on patient experience: number of comments and perceived usefulness from a hospital perspective. Int J Qual Health Care 24(5):509–516. 10.1093/intqhc/mzs039 10.1093/intqhc/mzs039 22833616

[45] Shapiro LM, Eppler SL, Roe AK, Morris A, Kamal RN (2020 Nov) The Patient Perspective on Patient-Reported Outcome Measures Following Elective Hand Surgery: A Convergent Mixed-Methods Analysis. J Hand Surg Am 9. 10.1016/j.jhsa.2020.09.008. S0363-5023(20)30538-4 10.1016/j.jhsa.2020.09.008 PMC8080672 33183858

[46] Lifland BE, Mangione-Smith R, Palermo TM, Rabbitts JA (2018) May-Jun;18(4):376–383 Agreement Between Parent Proxy Report and Child Self-Report of Pain Intensity and Health-Related Quality of Life After Surgery. Acad Pediatr. 10.1016/j.acap.2017.12.001 10.1016/j.acap.2017.12.001 PMC5936667 29229566

[47] Bevans KB, Riley AW, Landgraf JM (2017). Children’s family experiences: development of the PROMIS® pediatric family relationships measures. Qual Life Res.

[48] Detry MA, Ma Y (2016). Analyzing Repeated Measurements Using Mixed Models. JAMA.

[49] Mandrekar JN (2011). Measures of Interrater Agreement. J Thorac Oncol.

[50] Adachi T, Sunohara M, Enomoto K et al (2019 Mar-Apr) Japanese cross-cultural validation study of the Pain Stage of Change Questionnaire. Pain Rep 4(2):e711. 10.1097/PR9.0000000000000711 10.1097/PR9.0000000000000711 PMC6455691 31041416

[51] Cunningham NR, Kashikar-Zuck S, Mara C (2017). Development and validation of the self-reported PROMIS pediatric pain behavior item bank and short form scale. Pain.

[52] Rolstad S, Adler J, Rydén A (2011 Dec) Response burden and questionnaire length: is shorter better? A review and meta-analysis. Value Health 14(8):1101–1108. 10.1016/j.jval.2011.06.003 10.1016/j.jval.2011.06.003 22152180

[53] Galesic M, Bosnjak M (2009). Effects of Questionnaire Length on Participation and Indicators of Response Quality in a Web Survey. Public Opin Q.

[54] Sullivan M, Bornstein S (2016) The Effectiveness of Digital Surveys for Collecting Patient Feedback in Newfoundland and Labrador. The Newfoundland and Labrador Centre for Applied Health Research

