# Supplementary material for: The “STOP Pain” Questionnaire: using the Plan-Do-Study-Act model to implement a patient-family preferences-informed questionnaire into a pediatric transitional pain clinic
Source: J Patient Rep Outcomes. 2022 Nov 29;6:120. doi: 10.1186/s41687-022-00520-4 (PMC9708994; doi:10.1186/s41687-022-00520-4)
Supplement: Supplementary file 1 — Supplementary Material 1 [file 41687_2022_520_MOESM1_ESM.docx]

**Appendix. List of Thematic Categories**

| **Category** |
| --- |
| No Suggested Changes |
| No response |
| Unsure Respondent |
| Typographical Revisions |
| Repetition and Redundancy |
| Tailor Questionnaire to specific needs (Mobility and Pain Interference) |
| Tailor Questionnaire to specific needs (General) |
| Demographics |
| Tailor Questionnaire to specific needs (Sleep) |
| Already in the Questionnaire |
| Pain Description and Measurement |
| Medical History |
| Emotional State |
| Activities of Daily Life |
| Opposed to Questionnaires in General for Pain Assessment |
| Satisfaction Survey |
